# Mechanism and Data-Driven Grain Condition Information Perception Method for Comprehensive Grain Storage Monitoring

**DOI:** 10.3390/foods14193426

**Published:** 2025-10-05

**Authors:** Yunshandan Wu, Ji Zhang, Xinze Li, Yaqiu Zhang, Wenfu Wu, Yan Xu

**Affiliations:** 1 College of Biological and Agricultural Engineering, Jilin University, Changchun 130022, China; 2 Changchun Longjia Grain Storage Science and Technology Backyard, Changchun 130504, China; 3 School of Food and Strategic Reserves, Henan University of Technology, Zhengzhou 450001, China

**Keywords:** grain storage monitoring, Mechanism and Data Driven, numerical simulation, model initialization, holistic perception, intelligent grain management

## Abstract

Conventional grain monitoring systems often rely on isolated data points (e.g., point-based temperature measurements), limiting holistic condition assessment. This study proposes a novel Mechanism and Data Driven (MDD) framework that integrates physical mechanisms with real-time sensor data. The framework quantitatively analyzes solar radiation and external air temperature effects on silo boundaries and introduces a novel interpolation-optimized model parameter initialization technique to enable comprehensive grain condition perception. Rigorous multidimensional validation confirms the method’s accuracy: The novel initialization technique achieved high precision, demonstrating only 1.89% error in Day-2 low-temperature zone predictions (27.02 m^2^ measured vs. 26.52 m^2^ simulated). Temperature fields were accurately reconstructed (≤0.5 °C deviation in YOZ planes), capturing spatiotemporal dynamics with ≤0.45 m^2^ maximum low-temperature zone deviation. Cloud map comparisons showed superior simulation fidelity (SSIM > 0.97). Further analysis revealed a 22.97% reduction in total low-temperature zone area (XOZ plane), with Zone 1 (near south exterior wall) declining 27.64%, Zone 2 (center) 25.30%, and Zone 3 20.35%. For dynamic evolution patterns, high-temperature zones exhibit low moisture (<14%), while low-temperature zones retain elevated moisture (>14%). A strong positive correlation between temperature and relative humidity fields; temperature homogenization drives humidity uniformity. The framework enables holistic monitoring, providing actionable insights for smart ventilation control, condensation risk warnings, and mold prevention. It establishes a robust foundation for intelligent grain storage management, ultimately reducing post-harvest losses.

## 1. Introduction

Grain is not only a cornerstone of national economic development but also a vital resource for people’s livelihoods and strategic reserves, with direct implications for national security and social stability. In recent years, China has proposed reforms to optimize the central grain reserve management system, strengthen oversight of grain reserve operations, and minimize losses across the entire grain supply chain—from production and storage to transportation and processing [1,2]. At present, China primarily relies on grain storage as the key mechanism for balancing supply and demand in its food security system. The country maintains an extensive network of over 30,000 grain storage facilities, with typical storage cycles ranging from 3 to 5 years [3]. In 2024, China’s total grain output reached 706.5 Mt, surpassing the 700 Mt milestone, with an increase of 11.09 Mt compared to the previous year [4]. With national grain reserves approaching 700 Mt, China now accounts for over 70% of global grain storage capacity [4].

The safety of stored grain is a critical component of global food security, governed by a complex ecosystem influenced by both abiotic (temperature, humidity, moisture content, and porosity) and biotic factors (microorganisms and insect pests) [5,6]. Systematic monitoring of these parameters, particularly temperature, humidity, and moisture content, is essential for maintaining grain quality, as they serve as key indicators of ecosystem stability and storage safety [7]. Grain storage is unique due to its dynamic biological nature. As living organisms, grains exhibit nonlinear metabolic activities, including respiratory heating, fungal proliferation, and pest infestation [8,9]. These intrinsic properties present significant challenges for China’s storage systems, which must preserve grain quality across vast stockpiles and extended storage periods. Moreover, grain bulks function as large-scale granular systems with distinct thermal insulation properties. This structural characteristic leads to pronounced “time-lag effects” in heat and mass transfer processes, complicating the intelligent monitoring of storage conditions. Significant bottlenecks persist in the monitoring and sensing of core state parameters within grain bulks. Taking temperature monitoring as an example, most grain silos in China are equipped with sensor arrays, where real-time temperature data are acquired through pre-installed sensor cables. According to the industry standard LS/T 1813–2017 [10], these sensors must be deployed with horizontal spacing ≤ 5 m and vertical spacing ≤ 1.5 m. In practical applications, temperature and humidity sensors exhibit several technical limitations. Firstly, these sensors demonstrate localized sensitivity, with an effective monitoring radius of only 0.3–0.6 cm, making it difficult to achieve comprehensive global monitoring of internal conditions in grain piles. Secondly, mainstream resistive and capacitive relative humidity sensors exhibit significant temperature dependence, where their core parameters (resistance/capacitance values) drift with environmental temperature fluctuations, leading to systematic errors in humidity measurements [11]. Additionally, the complex cabling systems of these sensors are prone to aging, further exacerbating measurement accuracy degradation [12]. These factors collectively result in a fragmented understanding of internal grain pile conditions, insufficient real-time monitoring capabilities, and an inability to meet the dynamic and precise monitoring demands of grain storage processes. For moisture monitoring, moisture sensors or manual sampling are typically employed. However, moisture sensors suffer from insufficient accuracy, while manual sampling is labor-intensive, time-consuming, and poses a disruptive impact on the Stored Grain Ecosystem. Grain piles exhibit extremely low thermal conductivity, resulting in a severe time-lag effect that hampers real-time monitoring [13]. For example, if localized heating occurs between two horizontally placed sensors due to spoilage, heat propagation to adjacent sensors may take up to 2–3 weeks—far exceeding the one-week window before severe spoilage occurs [14,15]. In more extreme cases, when a heat source is 0.6 m away from a detection point, a temperature of 30 °C at the source requires approximately 640 h to raise the detection point’s temperature by just 1 °C [14]. This significant delay in heat and mass transfer severely compromises grain quality and safety, highlighting a critical limitation in current grain storage monitoring technologies.

In China’s national grain reserve monitoring systems, the non-holographic nature of stored-grain condition data poses a significant challenge, primarily manifested in the limitations of detection methods, research approaches, and the complexity of storage environmental factors. To compensate for unmonitored areas distant from temperature and humidity sensors, two main methods are currently employed. Data Driven Approach: This method relies on limited monitoring-point data and employs multi-field coupling theory of grain piles, combined with algorithms and modeling, to construct comprehensive cloud maps [16]. However, this approach heavily depends on sensor density, often leading to “partial representation of the whole” or “over-generalization” due to spatial variability. Significant temperature and humidity discrepancies may exist between monitoring points, making simple interpolation or coupling models inadequate for accurately reflecting overall conditions. Additionally, the lack of real-time data updates may render supplemented non-holographic data incapable of capturing dynamic changes, diminishing the practical utility of generated cloud maps. Mechanism Driven Approach: This method utilizes initial and boundary conditions of grain storage to establish the heat and mass transfer models, employing advanced computational modeling and simulation techniques-such as Finite Element Analysis (FEA), Computational Fluid Dynamics (CFD), and Finite Volume Method (FVM) to predict multi-parameter variations [17]. Nevertheless, this approach often suffers from weak correlation between computational models and real physical systems, resulting in low-fidelity and unreliable predictions, commonly described as “incomplete simulation” or “unrealistic simulation”. Model initialization is particularly challenging, requiring alignment between the simulation and the actual grain bin state at a selected time, with key parameter errors constrained within acceptable thresholds. However, due to the complexity of multi-field coupling models in grain piles, as well as external climatic disturbances and temperature fluctuations induced by pest and mold activity [18,19,20,21], ideal initialization is rarely achievable. Furthermore, simplifications and assumptions inherent in simulation models further degrade prediction accuracy. Within the existing simulation model framework, the primary approach to reducing discrepancies lies in adjusting the model’s tunable parameters. However, the multi-field coupled model of grain piles involves numerous interrelated parameters, among which temperature, moisture content, and humidity exhibit significant coupling relationships [5]. In practical monitoring, temperature data are generally more accurate, whereas humidity sensors are prone to measurement inaccuracies due to factors such as service time, thermo-hygrometric conditions, and foreign material adhesion [11,22]. Moisture data, on the other hand, are primarily obtained through sampling tests, which can only reflect localized conditions. Moreover, as storage duration increases, the initial average moisture content undergoes migration, rendering the detection results unrepresentative. Therefore, numerical simulation may serve as the most effective means to supplement non-holistic data.

The rapid advancement of artificial intelligence (AI) technology in recent years has introduced transformative opportunities for developing and implementing MDD approaches in the grain industry. This hybrid methodology synergistically combines mechanistic models with data-driven techniques, harnessing their complementary advantages to demonstrate remarkable potential in control optimization and intelligent decision-making applications. In grain drying control systems, researchers have successfully integrated mechanistic drying models with AI algorithms to optimize the comprehensive control processes of drying equipment. The innovative introduction of a “window”-based data-driven methodology has facilitated AI-powered autonomous learning capabilities, substantially elevating the intelligence level of drying operations [23,24]. For grain condition monitoring, scholars have employed mechanism-driven approaches to systematically analyze climatic patterns across China’s diverse geographical regions. By incorporating data-driven AI techniques and leveraging self-learning algorithms, they have achieved precise matching between various grain silo characteristics and measurement point configurations, significantly enhancing the accuracy of grain storage safety monitoring and early warning systems [16,25]. Regarding inventory supervision, researchers have developed sophisticated dynamic mechanistic models grounded in grain ecology theory and the multi-field coupling theory of grain piles. Utilizing deep neural network architectures, the research teams have trained these models on extensive datasets encompassing diverse grain temperature field scenarios, including empty silo conditions, newly stored grain parameters, ventilation states, partially filled silo configurations, and overheating situations [6,26,27,28]. This MDD AI analytical framework has successfully enabled intelligent detection and assessment of diverse grain storage states, establishing a robust technical foundation for early warning systems targeting abnormal storage conditions. These pioneering applications comprehensively demonstrate the significant potential of MDD approaches within the grain industry, while effectively facilitating deeper integration of AI technologies in agricultural applications. The methodology’s success in bridging theoretical models with empirical data patterns represents a substantial advancement in agricultural informatics, offering promising directions for future smart agriculture initiatives.

Based on this foundation, the purposes of this study included (1) proposing a Mechanism and Data Driven grain condition information perception method for comprehensive grain storage monitoring, which establishes a numerical simulation framework that integrates physical mechanisms with real-time monitoring data through a hybrid physics-informed and data-driven approach; (2) quantitatively analyzing the thermal transfer effects of solar radiation and external air temperature on silo boundaries; (3) developing a novel model parameter initialization technique based on interpolation optimization of monitoring data, which transforms discrete measurements into continuous field distributions through a multidimensional data-space mapping mechanism, enabling high-precision initial parameter assignment; (4) validating the method’s effectiveness and accuracy rigorously across multiple dimensions, demonstrating robust performance in practical applications; (5) further elucidating the dynamic evolution patterns of temperature, moisture, and humidity fields in grain during storage. These findings provide critical theoretical foundations and empirical data for intelligent storage environment regulation, particularly smart ventilation control, condensation risk warnings, and mold prevention strategies.

## 2. Materials and Methods

### 2.1. Data Sources

**Figure 1:**
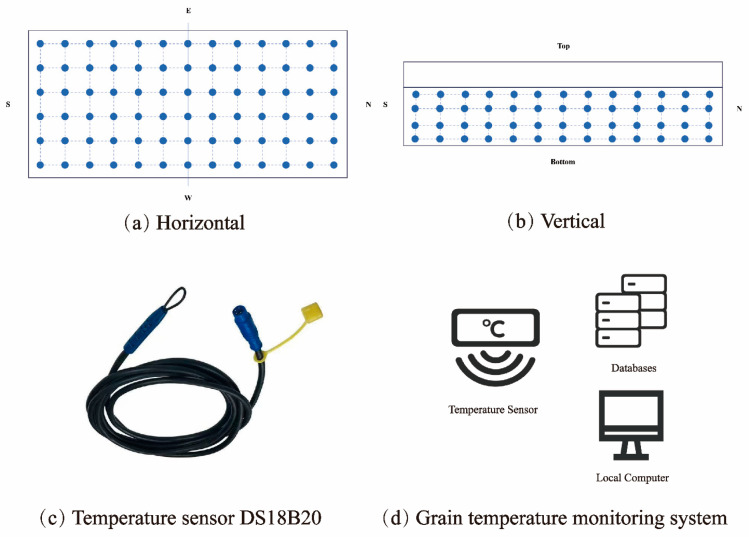
An example of a grain temperature monitoring system in a warehouse. (**a**) shows the horizontal arrangement of temperature sensors, while (**b**) demonstrates the vertical arrangement of temperature sensors. (**c**) presents the DS18B20 temperature sensor, which were utilized in the warehouse in this study, (**d**) illustrates an example of the grain temperature monitoring system.

Currently, most grain depots in China are equipped with comprehensive temperature monitoring systems. Temperature sensor cables are uniformly distributed throughout the granary, as shown in Figure 1a,b, to collect real-time temperature data during storage. The sensing system consists of 4 horizontal layers, with each layer containing 78 sensors. In most granaries, the temperature monitoring systems utilize the DS18B20 temperature sensor, which illustrates an example of a grain temperature monitoring system, as shown in Figure 1c,d. These data are then transmitted wirelessly to local computers or cloud databases for storage. Additionally, grain depot personnel conduct regular manual sampling to measure grain moisture content, and the results are manually recorded into computer systems or cloud databases to ensure data integrity and reliability.

This study selects a standard warehouse located in the Changchun region as the experimental subject. The granary represents typical storage conditions and is equipped with a well-established monitoring system, making it a representative example of warehouse granaries. The grain stored in this facility is maize, and the structural parameters of the granary are described in Table 1.

The research data were collected from 1 August to 30 August 2020, yielding a total of 9360 samples. This specific period was chosen based on the following considerations: (1) High-temperature risk period: August is characterized by high ambient temperatures, during which stored grain is particularly susceptible to thermal damage. The dataset captures critical dynamic changes in storage conditions effectively. (2) Data representativeness: The selected timeframe spans typical variations in grain storage environments, ensuring sufficient diversity for robust model training and validation.

During the data preprocessing phase, a rigorous data cleaning process was applied to the raw dataset. Obvious erroneous values (888 °C, −85 °C, and 85 °C) were identified as outliers and removed [29]. The processed dataset retains the essential characteristics of the original data while exhibiting significantly improved quality and reliability.

### 2.2. Mechanism Driven Model

#### 2.2.1. Mathematical Model

In accordance with the theory of heat and mass transfer in porous media, this study has established a coupled heat and moisture transfer dynamic model that incorporates the distribution of micro-airflow. During the development of this model, the following fundamental assumptions were invoked: (1) the airflow within the grain pile satisfies the incompressible condition; (2) the maize grain pile is treated as a continuous, isotropic, and homogeneous porous medium; (3) local thermal and moisture equilibrium conditions are maintained between the grain particles and the interstitial air [30,31,32]; and (4) exclude the grain’s autotrophic respiration. The governing equation system of the model, constructed in accordance with the three fundamental conservation laws, comprises: (i) the momentum conservation equation, which describes the airflow characteristics within the grain pile; (ii) the mass conservation equation, which characterizes the moisture migration processes; and (iii) the energy conservation equation, which governs the heat transfer mechanisms. This system of equations provides a comprehensive and mathematically robust framework for analyzing the coupled heat and mass transfer processes in the granary.

1. Continuity equation:

Assuming incompressible airflow within the grain pile with constant air density, the continuity equation derived from the mass conservation law is expressed as follows:

(1) $$\frac{\partial u_{j}}{\partial x_{j}}=0$$

where $u_{j}$ (*j* = 1, 2, 3) is the air flow velocity in the $x_{j}$ direction, m/s, $u_{1}=u$, $u_{2}=u_{3}=v$, and $x_{1}=x$, $x_{2}=y$, $x_{3}=z$ in the Cartesian coordinate system.

2. Momentum equation:

Since the velocity magnitude of natural convection is extremely low, the flow satisfies Darcy’s law. Assuming that the air density variation with temperature follows the Boussinesq approximation [33]. Based on the momentum conservation law, the governing equation can be expressed as follows:

(2) $$\rho_{a}\frac{\partial u_{i}}{\partial \tau }=-\frac{\partial P}{\partial x_{i}}+\delta_{ij}\rho_{0}g\beta \left(T-T_{0}\right)-\frac{\varphi \mu u_{i}}{K}$$

where $u_{i}$ represents the Darcy velocity in tensor form (m/s), τ denotes time (s), $\delta_{ij}$ is the intergranular void distance (mm), T indicates the grain pile temperature during storage (K), $T_{0}$ is the initial grain pile temperature (K), $\rho_{0}$ signifies the air density at temperature $T_{0}$ (kg/m^3^); φ corresponds to the equivalent diameter of rice grains (mm), β presents the thermal expansion coefficient.

3. Energy equation:

The heat transfer within the grain pile during natural storage satisfies the energy conservation principle. Accounting for grain respiration effects, the energy equation can be expressed as follows:

(3) $$\rho_{b}C_{b}\frac{\partial T}{\partial \tau }+\left(\rho_{a}C_{a}\right)u_{j}\frac{\partial T}{\partial x_{j}}=\frac{\partial }{\partial x_{j}}\left(k_{b}\frac{\partial T}{\partial x_{j}}\right)+h_{fg}\rho_{b}\frac{\partial W_{g}}{\partial \tau }$$

where $\rho_{b}C_{b}\frac{\partial T}{\partial \tau }$ represents the rate of heat change in the grain pile during natural storage, $\left(\rho_{a}C_{a}\right)u_{j}\frac{\partial T}{\partial x_{j}}$ denotes heat exchange caused by natural convection, $\frac{\partial }{\partial x_{j}}\left(k_{b}\frac{\partial T}{\partial x_{j}}\right)$, $h_{fg}\rho_{b}\frac{\partial W_{g}}{\partial \tau }$ indicates the heat of sorption/desorption during hermetic storage, $\rho_{b}$ is the density of paddy grains (${\mathrm{kg}/m}^{3}$), $C_{b}$ and $C_{a}$ are the specific heat capacities of grains and air, respectively (J/kg⋅K), $k_{b}$ is the thermal conductivity of grains (W/m⋅K).

Where the specific heat capacity $C_{b}$ and thermal conductivity of maize $k_{b}$ are related to the wet-basis moisture content of the grain bulk as follows [34]:

(4) $$c_{b}=1.465+0.0356M$$

(5) $$k_{b}=0.0654+0.0040T-3.6071\times 10^{-5}T^{2}$$

where M represents the wet-basis moisture content of the grain pile. The relationship between wet-basis and dry-basis $W_{g}$ moisture content is as follows:

(6) $$M=W_{g}/\left(1+W_{g}\right)\times 100$$

4. Mass conservation equation:

Based on the principle of mass conservation and accounting for grain respiration effects, the moisture conservation equation can be expressed as follows:

(7) $$\rho_{b}\frac{\partial W_{g}}{\partial \tau }+u_{j}\left(\frac{\sigma }{R_{v}T}\right)\frac{\partial W_{g}}{\partial x_{j}}=\frac{\partial }{\partial x_{j}}\left(D_{M}\frac{\partial W_{g}}{\partial x_{j}}\right)+\frac{\partial }{\partial x_{j}}\left(D_{T}\frac{\partial T}{\partial x_{j}}\right)-u_{j}\left(\frac{\omega }{R_{v}T}\right)\frac{\partial T}{\partial x_{j}}$$

where $\rho_{b}\frac{\partial W_{g}}{\partial \tau }$ represents the moisture variation rate; $u_{j}\left(\frac{\sigma }{R_{v}T}\right)\frac{\partial W_{g}}{\partial x_{j}}$ denotes moisture transport due to natural convection; $\frac{\partial }{\partial x_{j}}\left(D_{M}\frac{\partial W_{g}}{\partial x_{j}}\right)$ indicates moisture diffusion caused by moisture gradient, where $D_{M}=D_{eff}\sigma$ is moisture-driven water vapor diffusivity; $\frac{\partial }{\partial x_{j}}\left(D_{T}\frac{\partial T}{\partial x_{j}}\right)$ represents moisture diffusion driven by temperature gradient, where $D_{T}=D_{eff}\omega$ is thermal-driven water vapor diffusivity [35], $D_{eff}=\frac{D_{v}\varepsilon }{\tau_{b}R_{v}}$, $D_{v}=\frac{9.1\times 10^{-9}\cdot {\left(T\right)}^{2.5}}{\left(T+245.18\right)}$; $u_{j}\left(\frac{\omega }{R_{v}T}\right)\frac{\partial T}{\partial x_{j}}$ describes thermally induced moisture transport; $R_{v}=461.52J/\left(\mathrm{kg}\cdot K\right)$; σ denotes the change in water vapor partial pressure under constant temperature; ε is the porosity of the grain pile; $\tau_{b}$ represents the tortuosity of the grain pile; ω indicates the change in water vapor partial pressure at constant moisture content.

#### 2.2.2. Simulation Model

**Figure 2:**
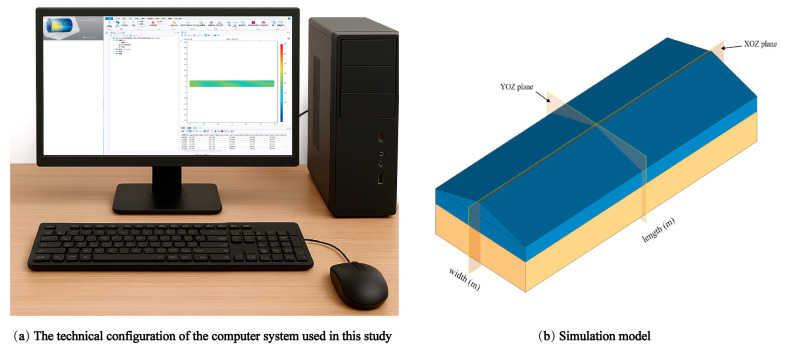
The technical equipment utilized in this study. (**a**) illustrates the technical configuration of the computer system used in this study. Numerical simulations were performed using COMSOL Multiphysics 5.6, which implemented a simulation model based on the actual grain bin structure (**b**), with key structural parameters detailed in Table 1.

Figure 2a illustrates the technical configuration of the computer system used in this study. The system runs Windows 11 on a Dell Precision 7920 Tower workstation with an Intel^®^ Xeon^®^ Silver 4214R CPU. To enhance computational performance, an NVIDIA RTX A4000 GPU was employed to accelerate numerical calculations and processing-intensive tasks. Numerical simulations were performed using COMSOL Multiphysics 5.6, which implemented a simulation model based on the actual grain bin structure (Figure 2b), with key structural parameters detailed in Table 1.

During the mesh generation process, the following optimization strategies were implemented to ensure computational accuracy. (1) Local Mesh Refinement: Finer mesh resolution was applied to the inner wall regions of the grain bin to better capture boundary effects. (2) Unstructured Grid Technology: This approach was employed to improve the model’s adaptability to complex geometries, ultimately achieving an average mesh quality of 0.9.

Table 2 presents the key physical properties of maize and air utilized in this study, providing a rigorous foundation for the material characteristics used in the numerical simulations. Through precise geometric reconstruction and optimized meshing, this modeling approach ensures the reliability and accuracy of the computational results, thereby enhancing the validity of the numerical analysis.

### 2.3. A Mechanism and Data-Driven Numerical Simulation Framework

This study innovatively proposes a Mechanism and Data-Driven Numerical Simulation Methods that address the limitations of traditional single-drive models by deeply integrating physical mechanisms and real-time monitoring data. The framework was developed through an in-depth analysis of existing modeling approaches, which were found to fall into two primary categories. Mechanism-driven models are grounded in physical laws, offering strong interpretability but constrained by theoretical assumptions. A typical example is the Heat and Mass Transfer Model [39,40,41]. In contrast, data-driven models rely on observational data, excelling at capturing nonlinear relationships but lacking a physical foundation. While these models effectively approximate real-world behavior, their mechanistic limitations can reduce reliability. Additionally, large datasets often suffer from quality issues, inefficient utilization, and the exclusion of low-probability events.

**Figure 3:**
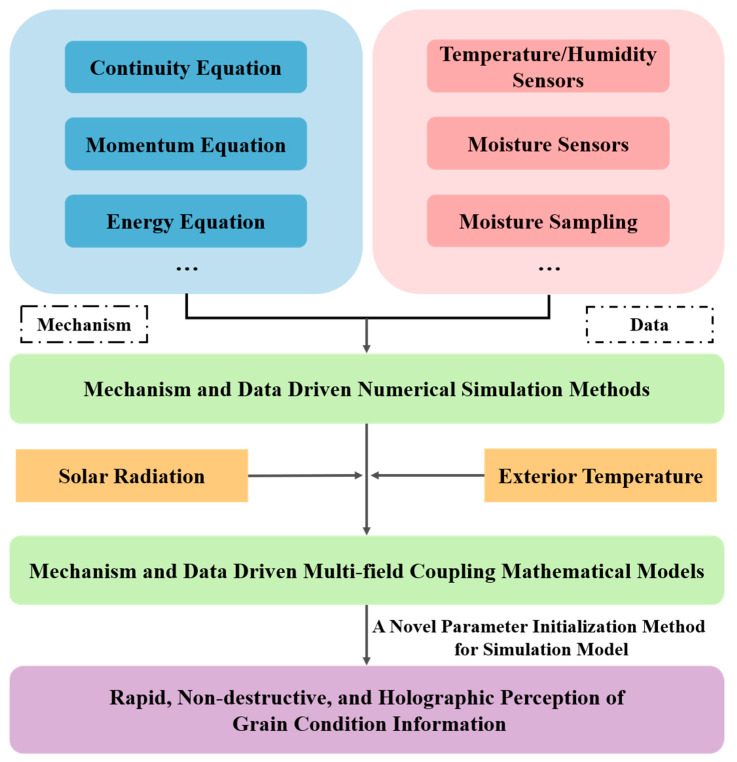
A framework for Mechanism and Data-Driven numerical simulation, incorporating three key components, Physical Equation Embedding, Data Driven Correction (including solar radiation and exterior temperature), and Initial Parameter Initialization.

The core innovation of this study lies in the establishment of a co-driven collaborative framework, which combines the strengths of both approaches. The framework incorporates three key components, Figure 3: (1) Physical Equation Embedding: This component preserves the theoretical foundation of mechanistic models by embedding physical equations into the numerical framework, ensuring a robust mathematical basis (Section 2.2.1 for details of the Mathematical Model). (2) Data-Driven Correction: This component introduces real-time monitoring data into the model by transforming it into dynamic boundary conditions. This approach quantitatively accounts for the effects of solar radiation and ambient temperature on thermal and moisture transfer within the storage structure. The integration of real-time data allows the mathematical model to dynamically adjust its parameters, enhancing its adaptability to real-world conditions. (3) Initial Parameter Initialization: This innovation moves beyond traditional empirical or mean-value-based parameter assignments. Instead, it integrates spatial interpolation techniques with deep learning algorithms to construct a multi-dimensional data mapping mechanism. This approach enables the conversion of discrete data into continuous-field representations, achieving highly precise initial parameter assignments that significantly improve simulation accuracy.

By integrating physical mechanisms with real-time data, this co-driven framework overcomes the limitations of traditional modeling approaches, offering a more robust and reliable methodology for numerical simulations.

### 2.4. Data Processing Methods

#### 2.4.1. Monitoring Data

In this study, a temperature field reconstruction method based on multi-step interpolation was implemented to achieve high-precision interpolation and visualization of temperature distributions using sensor monitoring data. After completing the data preprocessing, a Radial Basis Function (RBF)-based interpolation method was applied to the sensor data for temperature field reconstruction. The RBF interpolation method is distinguished by its high degree of smoothness and accuracy, enabling effective processing of non-uniformly distributed sensor data. During the interpolation process, the “cubic” method was selected as the interpolation kernel, and a fitting procedure was performed on the sensor coordinates and temperature values. For the temperature field visualization module, the Contour Plotter class from the Matplotlib (version 3.7.1) visualization library, implemented in Python 3.12, was utilized. This class employs a multi-tangent integral algorithm to generate high-quality contour plots, ensuring precise and clear visual representation of the temperature distribution.

#### 2.4.2. Simulating Data

This study utilized COMSOL Multiphysics software (version 5.6, developed by COMSOL Inc., Stockholm, Sweden) to perform numerical simulations. The numerical simulation results exported from COMSOL Multiphysics 5.6 encompass multiple physical field data, including temperature, relative humidity and moisture content. These datasets were converted into a storage format consistent with the experimental data acquisition system to ensure uniformity in subsequent processing workflows. During the data processing phase, the grid node temperature, humidity, moisture values, and velocity information extracted from the COMSOL Multiphysics 5.6 outputs were standardized according to the same format as the monitoring data. Corresponding contour plots for these variables were then generated. This meticulous processing workflow ensures not only the completeness and consistency of the simulation data but also provides high-quality data support for subsequent analysis and validation. The rigorous handling of these datasets guarantees their integrity and reliability, thereby establishing a robust foundation for further numerical simulations.

### 2.5. Model Validation Methods

**Figure 4:**
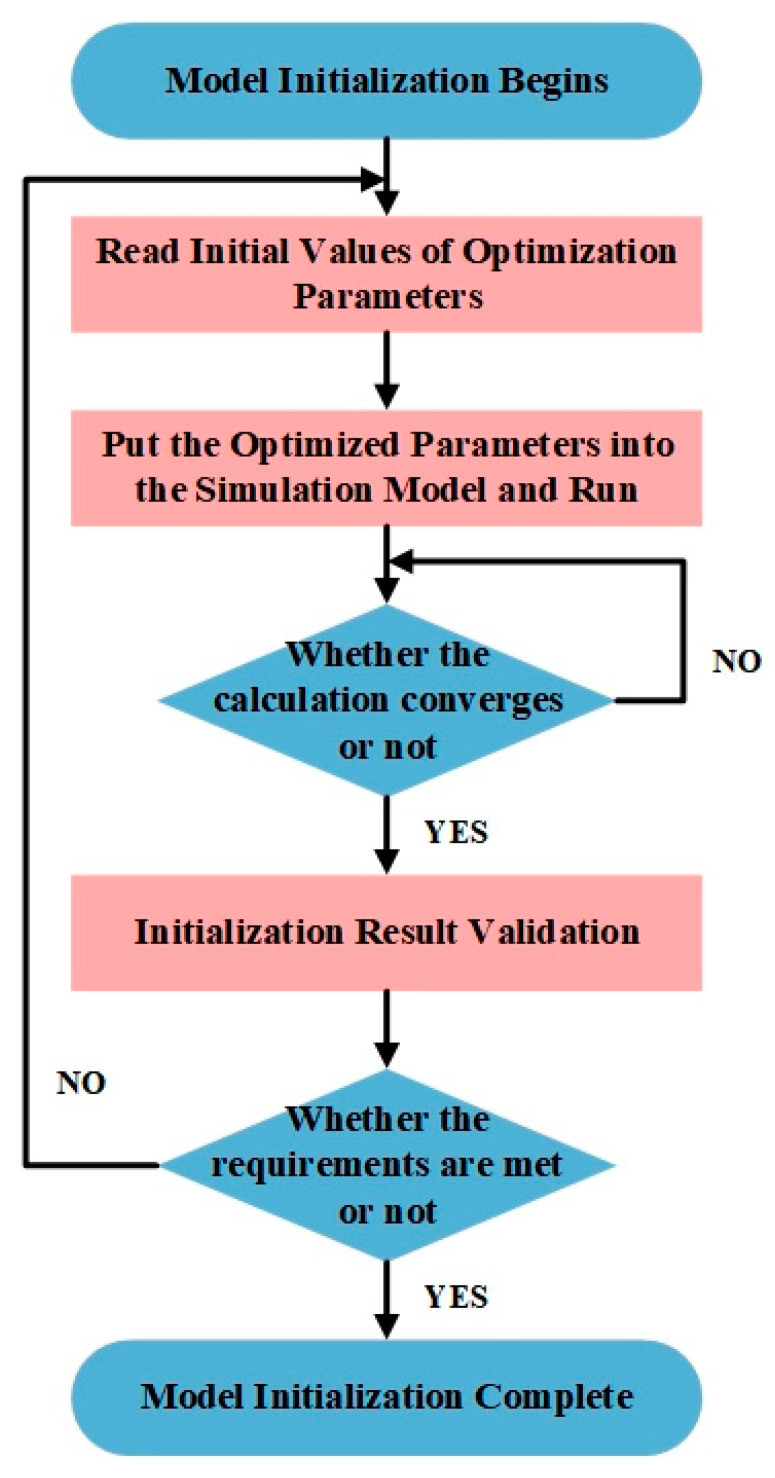
A novel parameter initialization process. During the model initialization phase, temperature field parameters were initially determined using an optimization algorithm. These parameters were then integrated into the models for iterative computation until successful convergence was achieved.

During the model initialization phase, the temperature field parameters were initially determined using an optimization algorithm, as depicted in Figure 4. These parameters were then integrated into a Mechanism and Data-Driven Multi-field Coupling Mathematical Models for iterative computation. The model’s convergence was evaluated based on a predefined criterion: if the computational results did not meet the convergence threshold, the parameters were adjusted, and the iterations continued until successful convergence was achieved. Following convergence, the initialization results underwent a comprehensive multidimensional validation. To ensure accuracy, three key characteristics of temperature distribution contour maps were utilized as validation metrics: (1) the number of low-temperature zones, (2) the spatial distribution area of these zones, and (3) the average temperature variation trend.

In this study, to quantitatively evaluate the quality of the monitored and simulated contour maps, we adopted two internationally recognized image quality assessment metrics: Peak Signal-to-Noise Ratio (PSNR) and Structural Similarity Index (SSIM). PSNR reflects the relationship between the image signal and noise by calculating the logarithmic value of the inverse of the Mean Squared Error (MSE), thereby quantifying the differences between pixels. On the other hand, SSIM evaluates the overall visual similarity of images by calculating their structural, brightness, and contrast similarities, which better aligns with human visual perception.

## 3. Results and Discussion

### 3.1. YOZ Plane-Model Validation Analysis

**Figure 5:**
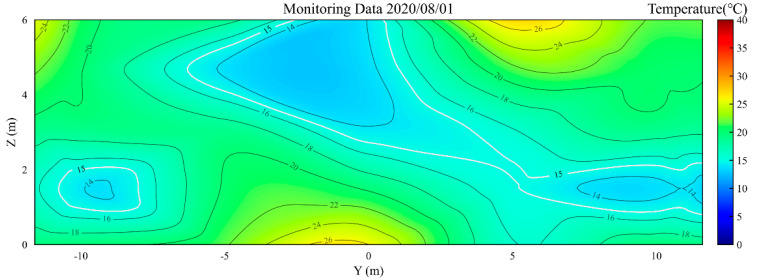
The temperature contour map of the YOZ plane during the simulation initialization on 1 August 2020.

Figure 5 presents the temperature contour map of the YOZ plane during the simulation initialization on 1 August 2020. As illustrated in Figure 5, three distinct low-temperature zones were observed in the grain pile during the initial storage phase. In this study, low-temperature zones are defined as localized regions where the internal temperature of the grain pile is significantly lower than the surrounding environment or other areas (specifically, below 15 °C), though the temperature difference does not meet the criteria for stable cold cores. The total area of the initial low-temperature zones was monitored as 29.67 m^2^ on 1 August 2020.

Table 3 presents the monitored and simulated total areas of low-temperature zones on the second day of model operation, which are 27.02 m^2^ and 26.51 m^2^, respectively, with an absolute error of 0.51 m^2^ (relative error of 1.89%). This demonstrates that the novel model parameter initialization method proposed in this study can achieve high-precision initial values, ensuring that the simulation model begins computations in a state highly consistent with the actual grain pile conditions.

**Figure 6:**
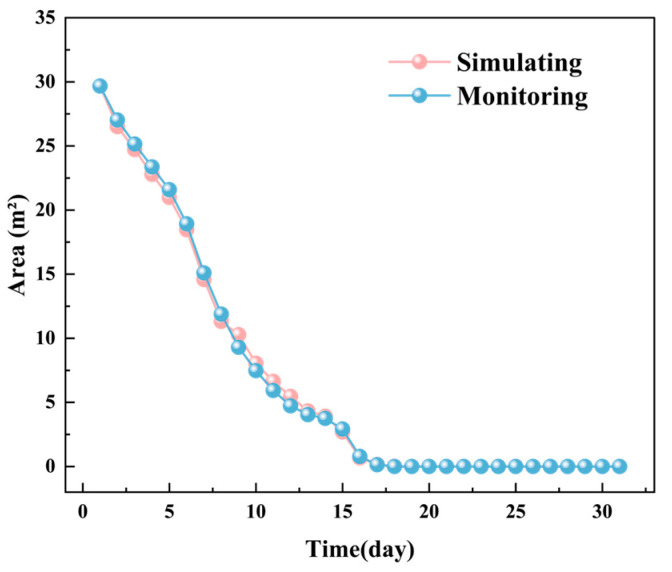
The monitored and simulated total areas of low-temperature zones in the YOZ plane of the grain pile over time.

**Figure 7:**
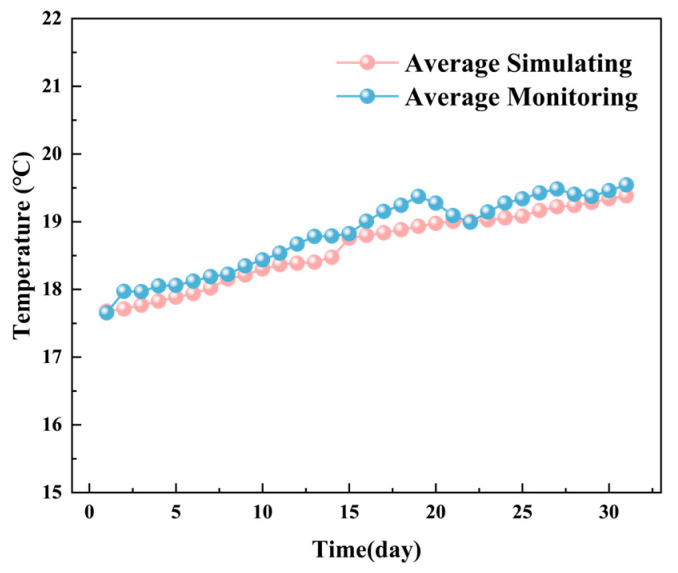
The monitored and simulated average temperature changes in the YOZ plane over time.

Figure 6 illustrates the monitored and simulated total areas of low-temperature zones in the YOZ plane of the grain pile over time, while Figure 7 shows the corresponding average temperature changes. The data indicate that the total area of low-temperature zones in the YOZ plane of the grain pile exhibits an increasing trend. The simulation results align closely with the actual monitored areas, with a maximum deviation of no more than 0.45 m^2^. Additionally, the average temperature in the YOZ plane demonstrates a monotonic increasing trend, with simulation results showing high consistency with actual monitored data (maximum deviation, <0.5 °C). This phenomenon is attributed to the intensified heat and moisture transfer processes within the grain pile due to the high temperatures in August [42].

**Figure 8:**
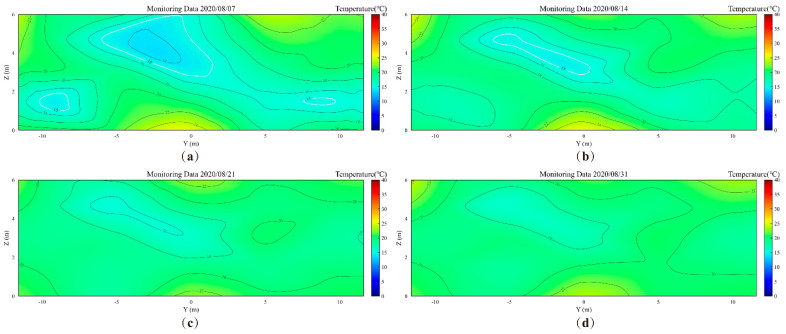
The monitored temperature contour maps of the grain pile’s YOZ plane. (**a**) 7th day; (**b**) 14th day; (**c**) 21st day, (**d**) 31st day.

**Figure 9:**
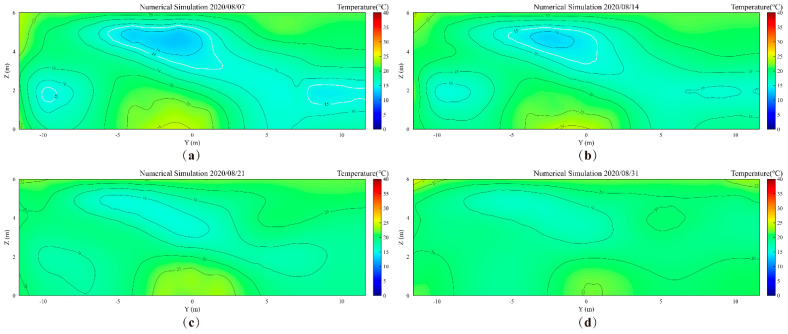
The simulated temperature contour maps of the grain pile’s YOZ plane. (**a**) 7th day; (**b**) 14th day; (**c**) 21st day, (**d**) 31st day.

Figure 8 and Figure 9 show the monitored and simulated temperature contour maps of the grain pile’s YOZ plane, respectively. Through analysis, it can be seen that by the 7th day of storage, there are three low-temperature zones in the YOZ plane, with monitored and simulated total low-temperature areas of 15.1 m^2^ and 14.58 m^2^, respectively, resulting in an absolute error of 0.52 m^2^. As the storage time progresses, by the 14th day, the monitored and simulated total low-temperature areas decrease to 3.75 m^2^ and 3.92 m^2^, respectively, with an absolute error of only 0.17 m^2^. Notably, by the 14th day of storage, the two low-temperature zones near the grain pile wall have completely disappeared in the monitored results. This phenomenon indicates that significant wall effects exist in the actual storage process, with external climatic conditions and solar radiation having a pronounced impact on the grain silo [33,42]. Additionally, the difference in the rate of low-temperature area decay between monitored and simulated values may stem from two aspects: first, the numerical model simplified the local bio-heat effects, and second, the numerical model has limitations in simulating complex nonlinear heat and mass transfer processes.

Further analysis of the comparison results (Figure 6, Figure 8 and Figure 9) reveals that the proposed numerical simulation framework accurately reproduces both the spatial distribution of low-temperature zones (including their number and locations) and their dynamic decay process over storage time. Specifically, by the 17th and 18th days of storage, the monitored results indicate that there are no low-temperature regions below 15 °C in the YOZ plane of the grain pile. However, the thermal inertia of the building envelope causes a significant time lag in the transmission of external disturbances through the envelope to the interior of the grain pile. This phenomenon results in a certain time difference between the numerical simulation results and the actual observed values, objectively verifying the important influence of the thermal buffering effect of the envelope structure on the evolution of the grain pile’s temperature field.

**Figure 10:**
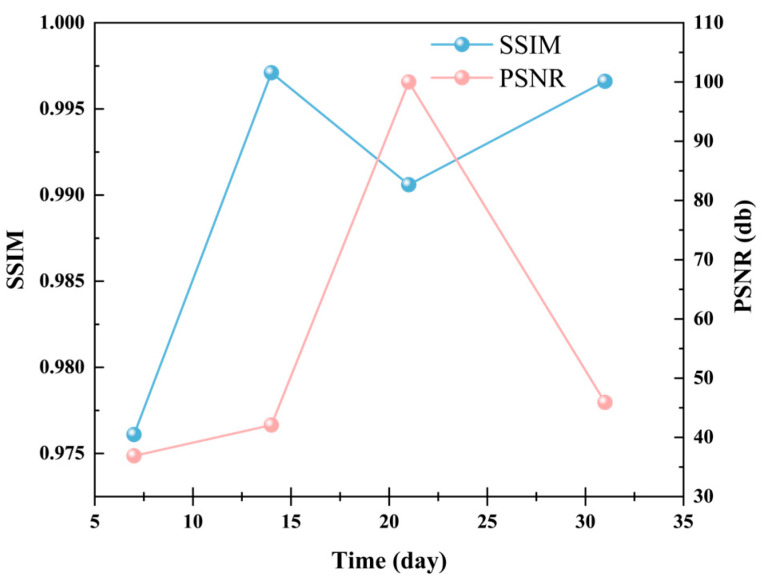
The similarity between monitored and simulated cloud maps. The SSIM values remain consistently above 0.97, indicating that the simulated contour maps exhibit high structural and visual similarity with the monitored contour maps.

To verify the quality of the simulated contour maps, we calculated the PSNR and SSIM values for monitored and simulated contour maps at different time points (7 days, 14 days, 21 days, and 31 days). As shown in Figure 10, the SSIM values consistently remain at 0.97 or higher, indicating that the simulated contour maps share high structural and visual similarity with the monitored contour maps. At 21 days, the PSNR value reaches infinity in theory, signifying that the simulated image is completely consistent with the monitored contour map at this time point. Meanwhile, at 31 days, the PSNR value achieves its highest level of 45.911 dB, and the SSIM value reaches 0.9966, further validating the superior performance of the simulated contour maps.

These results demonstrate that the simulated contour map generation method proposed in this study exhibits high consistency and superior generation quality at various time points, particularly reaching optimal levels in the later stages. This fully proves the validity of the mechanistic and data-driven simulation framework and models proposed in this study.

### 3.2. XOZ Plane- Multi-Field Interactions

After validating the feasibility and accuracy of the Mechanism and Data-Driven numerical simulation framework and models through temperature validation in Section 3.1, we have developed a novel approach for holistic grain condition perception. This approach involves using MDD numerical simulations to obtain key parameters such as grain temperature, moisture, and humidity. Taking the XOZ plane as an example, we subsequently analyze the complex multi-field interactions occurring during grain storage (Table S1).

#### 3.2.1. Temperature

At the initial stage of storage (1 August 2020), there are three distinct low-temperature zones inside the grain pile, with the following spatial distribution characteristics: (1) the middle-lower layer of the south exterior wall (Zone 1), (2) the core area of the grain pile (Zone 2), and (3) the middle-upper layer of the north exterior wall (Zone 3).

**Figure 11:**
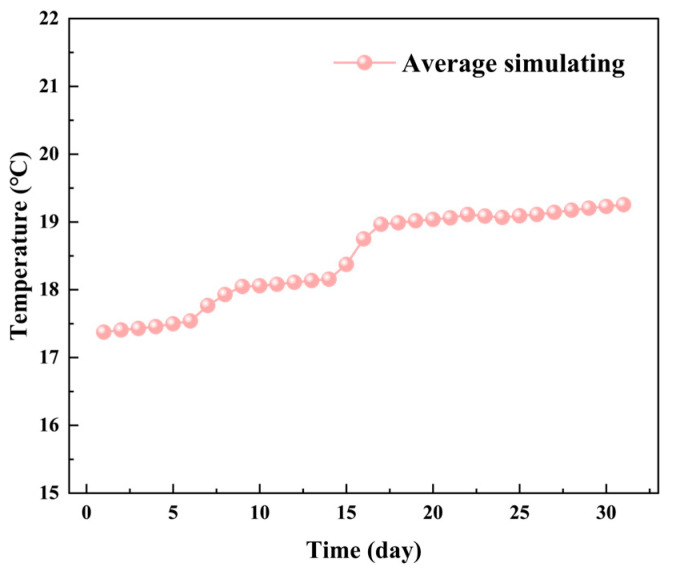
The trend of average temperature changes in the XOZ plane of the grain pile during one month of storage.

**Figure 12:**
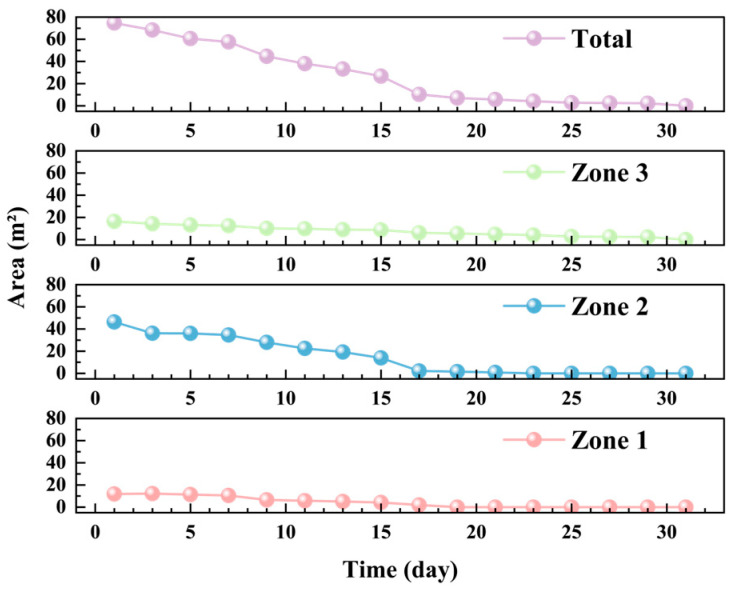
The trend of low-temperature zone area changes in the XOZ plane (zones 1, 2, 3, and total).

**Figure 13:**
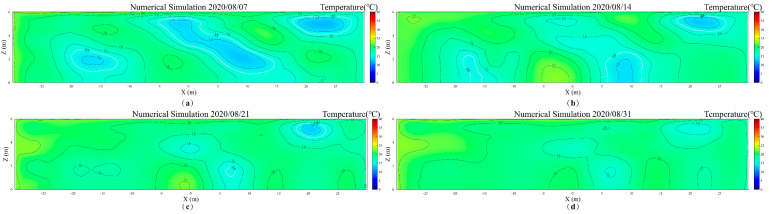
The temperature contour maps of the grain pile’s XOZ plane. (**a**) 7th day; (**b**) 14th day; (**c**) 21st day, (**d**) 31st day.

Figure 11 illustrates. The average temperature shows an increasing trend, similar to the average temperature changes observed in the YOZ plane. This is attributed to the high temperatures in August intensifying the heat and moisture transfer processes within the grain pile. Figure 12 presents the trend of low-temperature zone area changes in the XOZ plane during one month of storage, while Figure 13 shows the temperature distribution contour maps of the XOZ plane during the same period. By the 7th day of storage, the XOZ plane of the grain pile still exhibits three characteristic low-temperature zones. Compared to the initial values, the simulated total area of the low-temperature zones is reduced by 22.97%. Among these, the simulated area of Zone 2 is 34.65 m^2^ (a reduction of 25.30%), as this area initially occupied the largest proportion of the low-temperature zones. Its relatively large surface-to-volume ratio facilitated more significant heat and moisture exchange with the surrounding environment, leading to the most noticeable reduction in area. Meanwhile, Zone 1, located near the south exterior wall, shows a reduction of 27.64%, which is significantly higher than the 20.35% reduction observed for Zone 3. This discrepancy arises because Zone 1 is closer to the south exterior wall and is thus more strongly influenced by external climatic conditions.

The simulation results reveal significant differences in the disappearance times of the three low-temperature zones in the XOZ plane, Zone 1 disappears by the 19th day, Zone 2 by the 23rd day, and Zone 3, being the last, disappears by the 31st day. This phenomenon is closely related to the spatial conditions of each low-temperature zone. Specifically, Zone 1, adjacent to the south exterior wall, is directly exposed to significant external influences, whereas Zone 3, located near the north exterior wall and less affected by solar radiation, exhibits a delayed disappearance.

#### 3.2.2. Moisture

**Figure 14:**
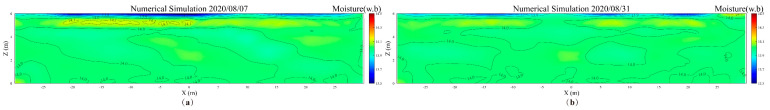
The moisture contour maps of the grain pile’s XOZ plane. (**a**) 7th day; (**b**) 31st day.

Figure 14a presents the moisture distribution characteristics in the XOZ plane of the grain bulk on the 7th day of storage. The results demonstrate a significant gradient decrease in moisture content at the surface layer, with the minimum value reaching 13.42% (w.b). This phenomenon results from the coupled mechanisms of thermodynamic effects and convective transport. The direct contact interface between the surface grain layer and warehouse air exhibits substantial differences in thermal properties. The thermal diffusivity of air (on the order of 10^−5^ m^2^/s) is two orders of magnitude higher than that of grain particles (on the order of 10^−7^ m^2^/s), making the surface layer more susceptible to external thermal disturbances [13,43]. Temperature gradient-induced air density differences drive natural convection, establishing a thermal circulation pattern (hot air rising, cold air sinking) that facilitates moisture migration toward middle and lower layers along the airflow path [44]. Notably, the heat and mass transfer within the grain bulk displays distinct spatial heterogeneity. The middle and lower layers, characterized by the low thermal diffusivity of grain particles and inherent thermal resistance, form an effective thermal buffer zone exhibiting significant thermal inertia.

Figure 14b further elucidates the moisture field evolution by the 31st day of storage. Compared to initial conditions, the upper-layer moisture shows continued thermodynamic-driven dissipation along the temperature gradient. Comparative analysis of temperature fields in Figure 13a,d reveals a characteristic anti-phase distribution in middle and lower layers: High-temperature zones correlate with low moisture content (<14%); Low-temperature zones accumulate higher moisture (>14%)

This spatiotemporal differentiation in thermo-hygroscopic coupling provides crucial theoretical support for the dynamic spatiotemporal regulation of intelligent aeration systems.

#### 3.2.3. Humidity

Based on the spatiotemporal evolution characteristics of moisture fields obtained through MDD numerical simulation, this study further elucidates the heat and mass coupling mechanisms within grain piles.

**Figure 15:**
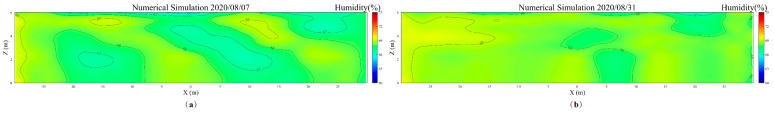
The humidity contour maps of the grain pile’s XOZ plane. (**a**) 7th day; (**b**) 31st day.

As demonstrated in Figure 15a, the XOZ plane analysis on day 7 reveals a strong positive correlation between relative humidity and temperature fields. The high-temperature zone exhibits a relative humidity of 70.15%, while the low-temperature region shows a reduced value of 66.5%. This coordinated distribution pattern stems from two fundamental mechanisms: (1) Enhanced vapor pressure effect: Elevated grain surface temperatures increase local vapor pressure, thereby raising micro-environment humidity levels [45]. (2) Thermally driven vapor migration: Water vapor transport through interstitial air spaces is governed by temperature gradients [46], establishing distinct thermo-hygroscopic coupling processes.

As illustrated in Figure 15b, the temperature field undergoes progressive homogenization Figure 13d, leading to corresponding uniformity in humidity distribution. Numerical simulations demonstrate that residual micro-airflows, sustained by lingering temperature gradients, facilitate continuous thermo-hygroscopic exchange and moisture redistribution. This dynamic equilibrium process mechanistically explains the mid-storage evolution of humidity fields. The quantitative findings provide critical theoretical support for: (1) Condensation risk early-warning systems-Predicting moisture accumulation zones. (2) Mold growth prevention strategies-Identifying microbial risk hotspots. (3) Storage environment optimization protocols-Guiding ventilation and climate control.

## 4. Discussion and Future Work

### 4.1. Comparative Analysis with Existing Methods

Comparative Analysis of Mechanism Driven, Data Driven, and MDD Framework Methods shows in Table 4. (1) Mechanism Driven Methods (Numerical Simulation) allow the acquisition of multi-parameter numerical values, such as temperature, humidity, and moisture. Wang, Y et al. reveal that simulated results exhibit a maximum deviation of 1.5 °C compared to experimental data when using mean initialization [47]. However, significant discrepancies arise due to environmental disturbances, leading to unreliable numerical results. Yin, J. further highlights that simulated values deviate from experimental values across different storage durations. Specifically, at t = 336 h, the deviation reaches 2.0 °C, while at t = 576 h, it reduces to 0.1 °C [36]. Initially, both the simulation and experimental warehouse exhibit uniform initial values. However, during storage, experimental conditions are influenced by external disturbances, whereas simulations lack real-time adjustments and interventions. This results in substantial differences between simulated and experimental results, rendering the numerical outcomes unreliable. (2) Data-Driven Methods are limited to obtaining only temperature data, which lacks comprehensive humidity and moisture information. Wu, W et al. propose a computer algorithm for monitoring stored grain using temperature data, achieving an average accuracy of 94% in identifying grain quality and inventory issues [16]. Li, X et al. demonstrate that the grain storage state classification model (3D DenseNet) achieves an accuracy of 97.38%, outperforming baseline models. Additionally, the temperature prediction model (3DCNN-LSTM) shows high accuracy, with MAE = 0.24 °C and RMSE = 0.28 °C [29]. (3) The MDD Framework in this study enables the acquisition of high-precision, holistic temperature, humidity, and moisture data. It employs a novel parameter initialization method, ensuring high-precision alignment with experimental or monitored conditions to minimize discrepancies. The maximum deviation between simulated and monitored data is 0.45 (representative plane), showcasing high accuracy. Furthermore, simulated results closely align with monitored data (<0.5 average temperature), validating the reliability of the simulation. The quality of simulated contour maps is thoroughly validated, with SSIM values above 0.97, confirming accuracy. However, the current model has certain limitations. For instance, it primarily focuses on corn, and while it has the potential to be adapted to other grain species, adjustments to physical properties such as thermal conductivity and density would be required. Additionally, the model currently focuses on flat warehouses, which are widely used in China and equipped with standardized temperature cables for grain monitoring. Future research will explore its application to silos and other warehouse types. The simulation duration is also limited to one month (8.1–8.31) due to computational constraints, though the model is capable of describing grain storage processes during other periods, such as colder winter months (e.g., 1.1–1.30). Future work will extend the simulation duration and validate the model across different time periods.

### 4.2. Future Development Plan

This research holds significant practical value as it addresses critical challenges in grain storage management by providing comprehensive insights into grain conditions. The ability to acquire holistic and accurate data on grain temperature, moisture, and storage conditions is indispensable for ensuring the stability and quality of stored grain. By implementing advanced monitoring and prediction systems, the study allows for the early identification of dangerous conditions such as heating, moisture accumulation, and mold growth. This early detection enables timely interventions, such as drying or cooling, to prevent spoilage and maintain grain quality [48]. For instance, if a potential heating zone is detected, the system can predict the required energy for drying operations, allowing for early resource allocation. The ability to predict energy requirements for drying or cooling operations allows for better resource planning and cost optimization. This proactive approach ensures that resources are used efficiently and effectively, reducing unnecessary expenses and enhancing the overall sustainability of grain storage management. The collection of precise and comprehensive temperature, humidity, and moisture data enables accurate predictions of grain quality parameters, such as fatty acid content, protein levels [49], and other critical indicators. These predictions are essential for maintaining the market value of grain and ensuring compliance with quality standards.

The implementation of this study is expected to yield substantial economic benefits for the agricultural and grain storage industries. These benefits include: (1) Reduction in Post-Harvest Losses: The ability to predict and mitigate risks such as heating, moisture accumulation, and mold growth will significantly reduce post-harvest losses, thereby increasing the overall yield of usable grain and enhancing food security. (2) Cost Savings Through Efficient Resource Utilization: By providing precise predictions of energy requirements for drying and cooling operations, the study ensures that resources are used optimally, leading to direct cost savings and improved operational efficiency. (3) Improved Grain Quality and Market Value: Accurate quality predictions enable the timely implementation of measures to preserve grain quality. This ensures that grain meets market standards, maintaining its value and preventing economic losses due to substandard quality.

To further advance this project and maximize its impact, we propose the following development plan: (1) Comprehensive Data Acquisition: Prioritize the collection of holistic data on grain piles, including temperature, moisture, fatty acid content, and other quality indicators. This will provide a detailed understanding of grain conditions and form the foundation for accurate predictions and interventions. (2) Advanced Predictive Modeling: Develop and refine short-term and long-term forecasting models to predict potential risks such as heating and moisture accumulation. For instance, if the system predicts a high-risk zone for heating, it can provide actionable insights into the energy requirements for drying, enabling timely resource allocation and intervention. (3) Integration of Real-Time Monitoring and Adaptive Systems: Enhance the system’s capability to perform real-time monitoring and adaptive adjustments. This will allow for dynamic responses to changing storage conditions, ensuring the system remains accurate and reliable over time.

## 5. Conclusions

This study proposed a novel Mechanism and Data-Driven grain condition information perception method for comprehensive grain storage monitoring, which established a numerical simulation framework that integrates physical mechanisms with real-time monitoring data through a hybrid physics-informed and data-driven approach. The study further quantitatively analyzed the thermal transfer effects of solar radiation and external air temperature on silo boundaries. The simulation models were initialized by the novel parameter initialization technique based on interpolation optimization of monitoring data, which transforms discrete measurements into continuous field distributions through a multidimensional data-space mapping mechanism. Comparative analysis demonstrates strong agreement between measured and simulated results: on the second day, the total low-temperature areas were 27.02 m^2^ (measured) and 26.52 m^2^ (simulated), with a relative error of just 1.89%, confirming the method’s high-precision initialization capability.

The method’s effectiveness and accuracy were validated rigorously across multiple dimensions, as shown in Table 5, demonstrating robust performance in practical applications. The simulated average temperature on the YOZ plane closely matched monitoring data (maximum deviation ≤ 0.5 °C), accurately reproducing both the spatial distribution (quantity/location) of low-temperature zones and their area attenuation dynamics during storage. The total area of low-temperature zones in the YOZ plane of the grain pile aligns closely with the actual monitored areas, with a maximum deviation of no more than 0.45 m^2^. Quantitative evaluation using PSNR and SSIM metrics showed consistently high SSIM values (>0.97), indicating excellent similarity between monitored and simulated cloud maps. Validation confirms the framework’s effectiveness, yielding a holistic grain condition information model.

This study further elucidates the dynamic evolution patterns of temperature, moisture, and humidity fields in grain during storage. Analysis of XOZ plane data demonstrated dynamic evolution patterns across temperature, moisture, and humidity fields. The observed average temperature increase aligned with the thermal trend previously identified in the YOZ plane. Compared to the initial values, the simulated total area of low-temperature zones decreased by 22.97%. Among these, Zone 2 (Center Area) showed a 25.30% reduction, while Zone 1 (near the south exterior wall) exhibited a more substantial decline of 27.64%, higher than the 20.35% reduction in Zone 3. Over time, Zone 1 disappeared by the 19th day, Zone 2 by the 23rd day, and Zone 3 (the most persistent) by the 31st day. High-temperature zones exhibit low moisture content, whereas low-temperature zones retain elevated moisture levels (>14%). A strong positive correlation exists between relative humidity and temperature fields. As the temperature field progressively homogenizes, it induces corresponding uniformity in humidity distribution.

These findings provide critical theoretical foundations and empirical data for intelligent storage environment regulation, including smart ventilation control, condensation risk prediction, and mold prevention strategies. They establish a robust data foundation and practical methodologies for intelligent storage management, ultimately reducing post-harvest grain losses.

## Figures and Tables

**Table 1:** The structural parameters of the warehouse, representing typical storage conditions and equipped with an advanced monitoring system, serve as a representative example of a grain storage facility.

Structural Parameters	Value/Feature
Warehouse type	Flat warehouse
Wall Thickness	50 cm
Eave Height (Exterior)	9.13 m
Eave Height (Interior)	9.0 m
Roof Thickness	20 cm
Grain Filling Height	6 m

**Table 2:** Key physical parameters of maize and air used in the numerical simulations, serve as a robust foundation for the material properties utilized in the study.

Parameter	Formulas/Values	Unit
Specific heat capacity of maize [34]	$c_{b}=1.465+0.0356M$	kJ/(kg⋅℃)
Thermal conductivity of maize [34]	$k_{b}=0.0654+0.0040T-3.6071\times 10^{-5}T^{2}$	W/(m⋅K)
Density of maize [34]	$\rho_{b}=735$	${\mathrm{kg}/m}^{3}$
Specific heat capacity of air [36]	$c_{a}=1.01+0.87T$	kJ/(kg⋅℃)
Thermal conductivity of air [36]	$k_{a}=0.0076T+0.02441$	W/(m⋅℃)
Density of air [36]	$\rho_{a}=1.205$	${\mathrm{kg}/m}^{3}$
Tortuosity [37]	$\tau_{b}=1.53$	-
Kinetic viscosity [37]	$\mu =1.79\times 10^{-5}$	Pa⋅s
Heat transfer coefficient of the warehouse wall [38]	$k_{\mathrm{wall}}=0.62$	${W/(m}^{2}\cdot ℃)$
Heat transfer coefficient of the warehouse roof [38]	$k_{\mathrm{roof}}=1.25$	${W/(m}^{2}\cdot ℃)$

**Table 3:** Total areas of low-temperature zones on the 2nd day (monitored vs. simulated).

Time (Day)	Different Approaches	Area (m^2^)
2nd day	Monitored	27.02
	Simulated	26.51

**Table 4:** Comparative Analysis of Mechanism Driven, Data Driven, and MDD Framework Methods (Accuracy/Reliability/Limitations).

Methods	Mechanism Driven Method	Data Driven Method	MDD Framework
Data Acquisition	Obtain multi-parameter numerical values (e.g., temperature, humidity, moisture)	Only obtain temperature data	Obtain high-precision, holistic temperature, humidity, and moisture data
Accuracy Reliability	A maximum deviation of 1.5 °C compared to experimental data (using mean initialization) [47].	Proposes a computer algorithm for monitoring stored grain using temperature data, achieving an average accuracy of 94% [16].	Employs a novel Parameter initialization method, ensuring high-precision alignment with experimental or monitored conditions to minimize discrepancies. The maximum deviation between simulated results and monitored data is 0.45 m^2^, demonstrating high accuracy. The simulated results align closely with monitored data (<0.5), validating the reliability of the simulation. The quality of simulated contour maps is thoroughly validated, with SSIM values above 0.97 confirming the accuracy.
	Simulated values deviate from experimental values across different storage durations. At t = 336 h, the deviation reaches 2.0 °C, while at t = 576 h, it reduces to 0.1 °C [36].	The grain storage state classification model achieves an accuracy of 97.38%, outperforming baseline models. The temperature prediction model (3DCNN-LSTM) demonstrates high accuracy with MAE = 0.24 °C and RMSE = 0.28 °C [29].	
Limitation	During the storage process, the experimental conditions are influenced by external disturbances, whereas the simulation lacks real-time adjustments and interventions. The substantial differences arise between the simulated and experimental results, leading to unreliable numerical simulation outcomes.	Can only obtain temperature data, which is limited and lacks comprehensive humidity and moisture information.	Grain Species: The model only focuses primarily on corn. Omission of Porosity: The model does not account for spatial variations in porosity. Limited Simulation Duration: The simulation is limited to a one-month period (8.1–8.31). Warehouse Types: The study primarily focuses on flat.

**Table 5:** The presentation of the performance and consistency of the simulation method across different dimensions further validates its effectiveness and reliability in practical applications.

Parameter	Statistical Metric	Maximum Deviation	Evaluation Description
Total Area of Low Temperature Zone in YOZ Plane	Total Area (m^2^)	≤0.45 m^2^	The maximum deviation between simulated results and monitored data is 0.45 m^2^, demonstrating high accuracy.
Average Temperature in YOZ Plane	Average Temperature (°C)	< 0.5 °C	The simulated results align closely with monitored data (<0.5), validating the reliability of the simulation.
Quality and similarity of Contour Maps	PSNR, SSIM Values	SSIM > 0.97	The quality of simulated contour maps is thoroughly validated, with SSIM values above 0.97 confirming the accuracy.

## Data Availability

The raw data supporting the conclusions of this article will be made available by the authors on request.

## Supplementary Materials

The following are available online at https://www.mdpi.com/article/10.3390/foods14193426/s1, Table S1: Temperature, humidity, and moisture data at the XOZ plane on 7 August and 31 August.
